# Potentially inappropriate prescribing in older adults in Mexico

**DOI:** 10.11606/s1518-8787.20210550033110

**Published:** 2021-11-12

**Authors:** Pedro Jesús Saturno-Hernández, Ofelia Poblano-Verástegui, Omar Acosta-Ruiz, Arturo Cuauhtémoc Bautista-Morales, Patricia María Gómez-Cortez, José Luis Alcántara-Zamora, Luis Miguel Gutiérrez-Robledo

**Affiliations:** I Centro de Investigación en Evaluación y Encuestas Instituto Nacional de Salud Pública Morelos México Centro de Investigación en Evaluación y Encuestas. Instituto Nacional de Salud Pública. Morelos, México; II Consorcio Mexicano de Hospitales Privados Ciudad de México México Consorcio Mexicano de Hospitales Privados. Ciudad de México, México; III Ciudad de México México Investigador independiente. Ciudad de México, México; IV Instituto Nacional de Geriatría Ciudad de México México Instituto Nacional de Geriatría. Ciudad de México, México

**Keywords:** Old person, Inappropriate Prescribing, Comorbidity, Misuse of Prescription Drugs, Medication-Related Side Effects and Adverse Reactions

## Abstract

**OBJECTIVE:**

To identify and quantify potentially inappropriate prescribing (*prescripción potencialmente inapropiada*, PPI) and other drug prescribing problems in public health care services in a population-based study at the three existing levels of complexity in Mexico.

**METHODS:**

Descriptive analysis of the Study on Satisfaction of Users of the Social Protection System in Health 2014–2016, prescription and drug supply section, to obtain the prevalence of PPI in older adults (≥ 65 years), based on Beers, STOPP, Prescrire and BSP listings using AM (older adults) prescription indicators, one for each listing.

**RESULTS:**

Most older adults (67%) were prescribed at least one medication, with a mean of 2.7 medications per prescription. The PPI prevalence was 74% according to the BSP criteria, 67% according to the STOPP listing, 59% with the Beer criteria, and 20% with Prescrire. The most frequent PPI prescriptions were NSAIDs, vasodilators and sulfonylureas.

**CONCLUSIONS:**

The use of PPIs in AM is high in Mexico. The higher prevalence found in this study may reflect the use of a source with population representativeness. The partial use and adaptations of the criteria make difficult comparing the studies; however, the STOPP criteria are the ones with the highest prevalence, as they cover a greater number of drugs and their use is more common in the first level of care.

## INTRODUCTION

Medication misuse in older adults (*adultos mayores*, AM) is recognized as a highly complex problem in the clinical practice. Some of the factors that contribute to this situation are the physiological changes associated with age, since they affect the pharmacodynamics and pharmacokinetics of some drugs, as well as the multiple morbidity that leads to varied pharmacological regimens. In addition, the aging of the population and health system factors also influence, as well as the limited accessibility and updating of guidelines for pharmacological consultation in AM; care with multiple specialists and frequently at different levels of complexity. This population group is very vulnerable to high-risk prescribing, including potentially inappropriate prescribing (PPI)^1,2^.

In AMs, high-risk prescribing is defined as the prescribing that may lead to adverse clinical outcomes, or that does not conform to the correct medication use. A variety of indicators have been proposed to quantify high-risk prescribing in AMs, including: polypharmacy, potentially inappropriate prescribing and exposure to high-risk medications^3^.

The PPI in the context of older people may be defined as the prescribing of “medications or classes of medications that generally should be avoided in persons aged ≥ 65 years because they are ineffective or pose an unnecessarily high risk to the elderly and a safer alternative is available”^4,5^.

Prescription in AM is an essential component in medical care and its correct application is a public health problem worldwide, since it is the population group of highest consumption and consequent increase in adverse events (AE) due to drug-drug interactions, which leads to clinical and economic impacts^6,7^.

PPI detection methods began in the 1990s. Beers was who firstly designed and published a detection tool, which consisted of an explicit list of drugs whose prescription, as identified by a group of experts, would be inappropriate. It was designed for AM residents of nursing homes specialized in older people’s care in the United States of America (USA). In 2012, an updated version was launched for use in outpatient care of the population ≥ 65 years. The update has a list of 53 groups of potentially inappropriate medications divided into three categories for AM: those to be avoided; to be avoided for given pathologies; and those to be used with caution^8^.

Some countries have gradually created or adapted the Beers list. A specific case is the STOPP criteria, developed in Europe by experts in geriatric pharmacotherapy for AM living in the community. It includes greater number of groups of potentially inappropriate drugs (65), divided by devices and systems. In addition, they consider other criteria such as comorbidity, severity of the pathology for which the drug is indicated and duration of treatment^9^.

France developed its own criteria, called “Prescrire”. It is a list of 74 groups of drugs that are more harmful than beneficial in all indications, i.e., for all age groups and general population. These criteria were established by systematic literature search rather than using a panel of experts^10^.

In developed countries, 20% of the population is ≥ 60 years old, and it is estimated to be 32% by 2050. In developing countries, the ratio of the population aged 60 years or older is expected to increase from 8% in 2005 to 20% by 2050^6^.

In a middle-income country such as Mexico, the population aged ≥ 65 years in 2019 was estimated to be 9.5 million, accounting for 7.5% of the total population. In 2030, AMs will be 14 million (10.2% of the total population)^11^.

The main causes of death among AMs in Mexico during 2017 were cardiovascular diseases (27.1%); diabetes mellitus (16.6%); chronic degenerative diseases, such as malignant tumors (11.6%), and cerebrovascular diseases (6.7%)^12^.

Using the Beers criteria in the USA, a prevalence of PPI in outpatient AM was found to be 25–30%, and 40% in homes for older people (2009 and 2012)^2,8^. Similar figures were reported in Ireland in 2008, 25% using the Beers criteria and 35% using the STOPP criteria^9^. Few studies have been conducted in Mexico. In 2014 a study of prevalence of PPI among AM of ≥ 70 using the Beers criteria found prevalence levels of 49% in a public hospital. However, each prevalence reported should carefully considered, due to the high variability of the samples selected, as well as the partial use of the components of each criterion for measuring^13^.

The use of inappropriate medication is a major concern in patient safety, especially for the AM population, due to its characteristics, as previously mentioned^6^. PPI has important impacts on several healthcare areas. Studies have been carried out to investigate how the identification of PPI among AM can reduce the burden of AE, hospitalization and drug-related mortality^1,2,5^. In addition, it has been estimated that PPI-related annual health expenditure is 7.2 billion dollars in the USA^8^.

Mexico has an Essential Drug List (EDL)^14^ that serves as a guide for all public healthcare institutions, based on the concept of rational drug use proposed by the World Health Organization (WHO): efficacy, safety and cost. However, there are few studies on drug prescription patterns and errors among AM in Mexico, and even fewer in public primary healthcare or outpatient care institutions^6^. In the last five years, two studies were conducted in primary healthcare units of the Mexican Social Security Institute, which is one of the main healthcare institutions in Mexico that serves the social security-dependent population. In both studies, the populations reported were small^15,16^. Some of the findings of these studies are as follows: the use of non-steroidal anti-inflammatory drugs in hypertensive patients, with peptic acid disease or for a period longer than three months; use of glibenclamide that increases the risk of hypoglycemia; use of benzodiazepines with risk of balance disorders; use of calcium-antagonists in patients with constipation; use of diuretics in patients without congestive heart failure; β-blockers combined with verapamil or in the presence of chronic obstructive disease, where β-blockers can alter the ventilatory dynamics.

It is necessary to dimension at population level the issue of inappropriate drugs prescription, review its prevalence, and the drugs that are most frequently prescribed in an inadequate way. In this way, it would be possible to reduce the risk of events associated with inappropriate prescribing, reduce costs and provide safer and more effective care. The objective of this study is to identify and quantify PPI and other issues related to the prescription of medications in public healthcare services in a population-based study at the three levels of complexity existing in Mexico.

## METHODS

A descriptive cross-sectional study was performed on the Study of Satisfaction of Users of the Social Protection System in Health (ESASPSS) applied in 2014, 2015 and 2016. The general objective of the ESASPSS was to document the perception of satisfaction of SPSS users with the health services they received and, in due course, make recommendations to increase user’s satisfaction, and influence the effective access to healthcare services.

The survey was probabilistic, stratified and two-stage, with national, state and regional representativeness. The medical units attended by the users were defined as primary sample units, the approximate number of units per state was 26 totaling approximately 832 units in the country, distributed among the three levels of care: 80% first level, 16% second level and 4% third level. For each unit selected, 31 questionnaires were applied, estimating an effective sample of around 26,000 interviews nationwide per year. The detailed description of the sampling procedure, and the survey methodology are described in a previous publication^17^.

This study used the section of drugs prescription and supply of the ESASPSS for the three years. The section contains information on the drugs recorded in users’ prescriptions. The survey sampling comprised AMs aged ≥ 65 years who attended the healthcare facilities and were prescribed at least one medication on the day of the survey. The AMs who attended for reasons other than medical consultation were excluded; a final subsample of 6,071 AMs accrued over the three years was obtained.

The Beers^8^, STOPP^9^ and Prescrire^10^ lists were used to identify the PPI. The 2012 update of the Beers criteria was used; none of the 53 groups of drugs included in this criterion were excluded. For the STOPP criteria, the 2008 list was used. For Prescrire, the 2016 version of 74 drugs was used. From the three criteria, a criterion called “BSP” was created. In it the PPI is considered in cases where at least one contraindicated drug was found in any of the lists. Some contraindicated drugs appear in two or more lists of the above mentioned criteria.

For the four criteria, all listed drugs were considered regardless of the diagnosis or condition of each individual in the database.

### Construction and Analysis of Indicators

Four indicators were constructed for the analysis: two general indicators of prescribing among AM, one on the percentage of PPI according to each criterion used and, finally, one that evaluates the most frequent groups of contraindicated drugs (Box).

**Box:** Description of indicators of contraindicated drugs in older adults (AM).

Indicator	Formula	Description
**I. General indicators of prescriptions in AM**^a^		
Percentage of AM users with prescription	$\frac{\Sigma \;\mathrm{AM}\;\mathrm{with}\;\mathrm{prescription}}{\Sigma \;\mathrm{Total}\;\mathrm{AM}}$	Percentage of AMs who were prescribed pharmacological treatment.
Average number of medications in the AM’s prescription.	$\frac{\Sigma \;\mathrm{Medications}\;\mathrm{prescribed}\;\mathrm{to}\;\mathrm{the}\;\mathrm{AM}}{\Sigma \;\mathrm{AM}\;\mathrm{with}\;\mathrm{prescription}}$	The average number of medications on prescriptions. This indicator allows us to know the polypharmacy degree existing in prescriptions in *Seguro Popular* units.
**II. Percentage of contraindicated drugs according to criteria**^b^		
Percentage of contraindicated drugs prescribed in AM	$\frac{\Sigma \;\mathrm{Contraindicated}\;\mathrm{drugs}\;\mathrm{in}\;\mathrm{AM}}{\Sigma \;\mathrm{Drugs}\;\mathrm{in}\;\mathrm{AM}\;\mathrm{prescription}}$	According to the classification, it refers to the contraindicated drugs prescribed divided by the number of drugs in the prescription; if the value is close to 1, it means that most of the drugs in the prescription are contraindicated; if it tends to 0, it means that it is the lowest percentage of contraindicated drugs.
**III. Most frequent group of contraindicated drugs prescribed to AM**		
Percentage of the drugs most prescribed in the contraindicated	$\frac{\Sigma \;\mathrm{Contraindicated}\;\mathrm{group}\;\mathrm{in}\;\mathrm{AM}}{\Sigma \;\mathrm{Medications}\;\mathrm{contraindicated}\;\mathrm{in}\;\mathrm{AM}}$	Drugs are categorized according to the type of drug, and divided by total contraindicated drugs.

^a^ The indicator is calculated stratifying by older adult population and non-older adult population, for each year the survey was conducted.

^b^ The estimator is calculated in the same way for each of the criteria (Beers, STOPP, Prescrire and BSP), stratifying by the number of drugs prescribed to the user in the prescription.

To apply the PPI indicator by criterion, the drugs prescribed for each user were scored and coded, generating binomial variables by criterion, with a value of 1 = contraindicated and 0 = not contraindicated. The procedure and formulas used for each indicator are detailed in the Box.

To compare percentages between the survey years, Pearson’s chi-squared test was used, taking a value of p ≤ 0.05 as significant. Additionally, the indicator was analyzed by geographic area, creating four maps of the Mexican Republic (one for each criterion), where the prevalence of PPI was ranked. To assign the tonality in all the maps, these were categorized into 20% quintiles according to the range (maximum minus minimum) of each criterion.

Finally, to determine the main groups of PPIs, these were grouped according to the active substance listed on the US Food and Drug Administration website^18^. For the distribution of groups of drugs, they were recorded according to the active substance contained, and divided by the group of drugs prescribed among AM, with their respective 95% confidence interval (95%CI).

All statistical calculations were adjusted for survey design, considering weightings to obtain population estimators.

## RESULTS

Of the total AMs in the three years of study (6,071), on average, 67% of AMs were prescribed at least one medication. The year 2014 showed the highest prescription prevalence, 71%, with a decrease of 13 percentage points in 2016. The mean number of medications per prescription was 2.7 (95%CI 2.7, 2.8), the difference by year was only significant in 2016 with the lowest mean of 2.4 medications per prescription (Table 1).

**Table 1:** Results of general indicators of prescriptions in older adults (AM), according to the year of the survey.

Year	2014	2015	2016	Total
**Total AM in the survey**	**2,814**	**2,023**	**1,234**	**6,071**
**Total drugs prescribed among AM**	**1,996**	**1,352**	**710**	**4,058**
Percentage of AM with prescription (95%CI)	70.8 (69.1–72.5)	66.8 (64.8–68.9)	57.4 (54.6–60.1)	66.8 (65.6–67.9)
Average number of medications in the AM’s prescription. (95%CI)	2.7 (2.6–2.8)	2.9 (2.8–2.9)	2.4 (2.3–2.5)	2.7 (2.7–2.8)
Percentage of contraindicated drugs among AM according to Beers criteria^a^ (95%CI)	60.6 (58.5–62.8)	60.2 (57.6–62.8)	53.8 (50.1–57.5)	59.3 (57.8–60.8)
Percentage of contraindicated drugs among AM according to STOPP criteria^a^ (95%CI)	69.6 (67.6–71.6)	68.3 (65.9–70.8)	57.0 (53.4–60.7)	67.0 (65.5–68.4)
Percentage of contraindicated drugs among AM according to Prescrire criteria^a^ (95%CI)	19.8 (18.1–21.6)	22.7 (20.5–24.9)	15.2 (12.6–17.9)	20.0 (18.8–21.2)
Percentage of contraindicated drugs among AM according to BSP* criterion (95%CI)	**76.3 (74.4–78.2)**	**76.4 (74.1–78.7)**	**64.6 (61.1–68.2)**	**74.3 (73.0–75.6)**

^a^ Percentage calculated over the total number of drugs prescribed among AM.

Table 1 shows the prevalence of PPI in AM by each criterion. The highest prevalence was found using the criterion BSP created for the study, with 74% (95%CI 73.0, 75.6). This behavior occurred in the three years of the study. Of the criteria used internationally, the STOPP reported the highest prevalence, 67% on average, while the lowest prevalence was Prescrire, 20% on average. In 2016, the lowest figures were presented for the four criteria used in comparison to previous years.

**Figure 1:**
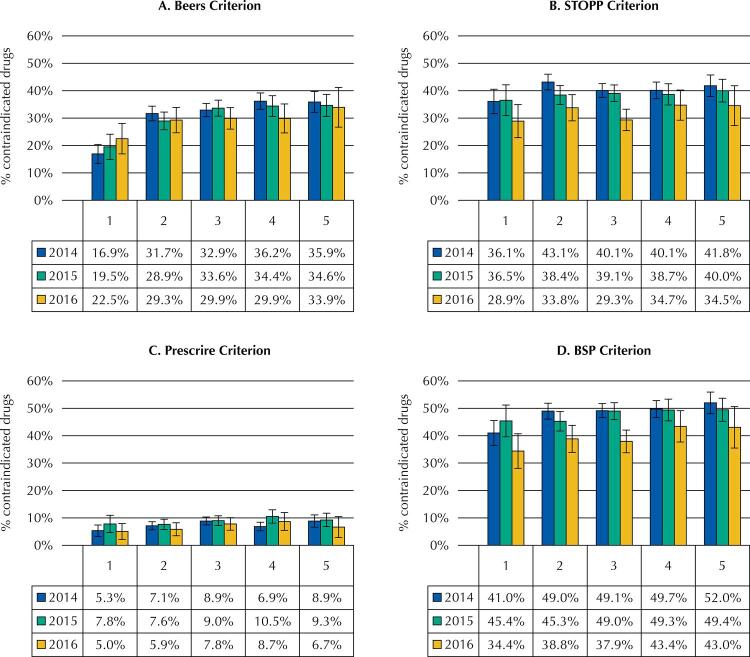
Contraindicated medications according to number of medications in the prescription, by criterion and year.

The prevalence of PPI does not disclose significant differences between years of measurement and criteria used, but an increase in prevalence is observed as the number of drugs prescribed increases (Figure 1). In the Beers, STOPP and BSP criteria, the highest prevalence occurred when 7 drugs were prescribed in the same prescription (29.6%, 32.6% and 44.3% on average, respectively).

**Figure 2:**
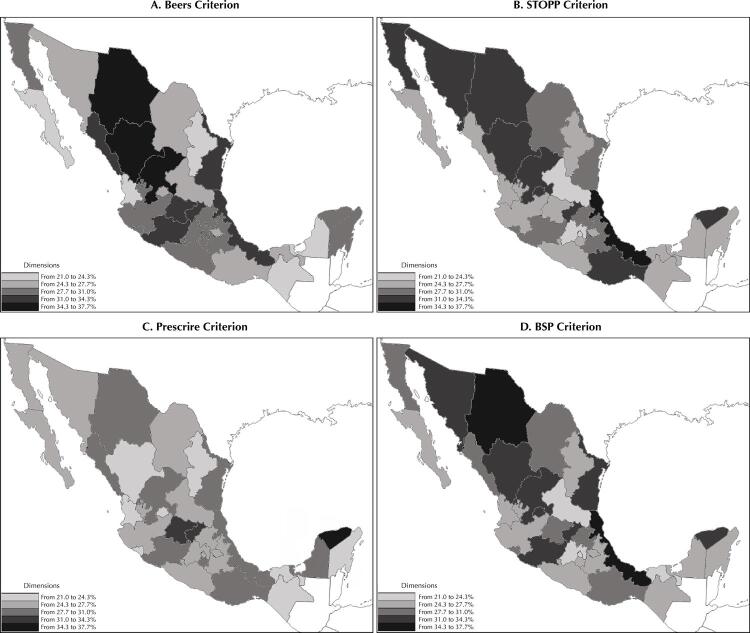
Percentage of contraindicated medications prescribed in older adults by federal entity in the years 2014–2016.

Figure 2 shows the regions of the country with highest prevalence of PPI. The highest percentages using the Beers, STOPP and BSP criteria prevail in the north; Chihuahua, Durango and Zacatecas account for the highest ones. In the remaining country, there is variability among criteria. In the southern region, Veracruz and Yucatan reach prevalence levels similar to those observed in the northern states.

The NSAIDs are the group of PPIs most prescribed in the three-year sample (25%, 36% and 32% respectively). Ranked in second there is a group of oral hypoglycemic agents (sulfonylureas), followed by drugs for cardiovascular pathologies such as: vasodilators, beta-blockers, diuretics and calcium blockers (Table 2).

**Table 2:** Groups of contraindicated drugs most frequentlya prescribed in older adults (MA).

2014		2015		2016		2014–2016	
**Type of drug**	**n = 2,539**	**Type of drug**	**n = 1,696**	**Type of drug**	**n = 673**	**Type of drug**	**n = 4,908**
NSAIDS	25.0% (23.4–26.7)	NSAIDS	26.4% (24.3–28.5)	NSAIDS	31.8% (28.3–35.3)	NSAIDS	26.4% (25.2–27.7)
Vasodilators	13.7% (12.3–15.0)	Vasodilators	11.1% (9.6–12.6)	Sulfonylureas	8.8% (6.6–10.9)	Vasodilators	12.0% (11.1–12.9)
Sulfonylureas	12.8% (11.5–14.1)	Sulfonylureas	10.8% (9.4–12.3)	Diuretics	8.3% (6.2–10.4)	Sulfonylureas	11.6% (10.7–12.4)
Diuretics	9.4% (8.3–10.5)	Diuretics	9.1% (7.7–10.4)	Proton pump inhibitors	7.7% (5.7–9.7)	Diuretics	9.1% (8.3–10.0)
Beta-blockers	8.5% (7.4–9.6)	Beta-blockers	9.0% (7.6–10.3)	Vasodilators	7.7% (5.7–9.7)	Beta-blockers	8.5% (7.7–9.2)
Calcium channel blockers	7.0% (6.0–8.0)	Calcium channel blockers	7.1% (5.9–8.4)	Beta-blockers	7.0% (5.1–8.9)	Calcium channel blockers	7.0% (6.3–7.7)
Proton pump inhibitors	5.8% (4.9–6.7)	Proton pump inhibitors	5.4% (4.3–6.5)	Calcium channel blockers	6.8% (4.9–8.7)	Proton pump inhibitors	5.9% (5.3–6.6)
Fibrates	3.3% (2.6–4.0)	Fibrates	4.4% (3.4–5.3)	Fibrates	4.6% (3.0–6.2)	Fibrates	3.8% (3.3–4.4)
H2-receptor antagonist	2.8% (2.2–3.5)	H2-receptor antagonist	3.1% (2.2–3.9)	Corticosteroids	2.2% (1.1–3.3)	H2-receptor antagonist	2.8% (2.3–3.3)
Antihistamines	2.4% (1.8–3.0)	Antihistamines	2.2% (1.5–2.9)	H2-receptor antagonist	2.1% (1.0–3.2)	Antihistamines	2.3% (1.9–2.7)

^a^ Values in percent (95%CI).

## DISCUSSION

The results of this study show relevant aspects regarding the quality of healthcare services provided to the population aged ≥ 65 years, particularly in relation to drugs prescribing. Considered by type of criteria used, there are differences by region, type of drug and number of drugs prescribed. Because the largest proportion of the sample is represented by first-level care medical units, results show mainly the behavior of these units.

The number of drugs per prescription is below that found by the WHO (3.5) in the same age group of the population^19^. Despite the lower number of drugs, the prevalence of PPI found in the study is similar to that reported by community studies under the STOPP criteria conducted in Canada, Europe, Australia and Asia (from 21% to 69%)^20^. The very wide range between prevalence levels may be due to the indiscriminate use of multiple criteria, adaptations, and the source of information used for their application, especially information on dosage and the study population’s history of pathologies. This hinders comparability between studies.

In 2014, population-based studies conducted in Ireland comprising AM aged ≥ 65 years found prevalence levels of 15%, taking into account the morbidity and dosage of the drugs, and 36% taking only the presence of the contraindicated drug. The STOPP criteria were used for both studies, and the sample was similar to the current study^21^. The prevalence levels of previous studies are twice lower than those found in this study. This difference may be a result of the use of comorbidity for the identification of PPIs. Simplifying criteria disregarding this variable allows a greater number of drugs to be considered PPIs, increasing prevalence levels. In a study carried out in a primary healthcare unit in Mexico, in which morbidity was considered, a prevalence of 67% was found^15^, the same as that found in this study. Therefore, it could be inferred that the prevalence of PPI in Mexico is high.

In Latin America, specifically Colombia and Argentina, lower prevalence levels have been found (21% and 20%, respectively) using the Beers criteria. These studies only considered outpatient care at the first level of care in AM. Our results are more representative of the first level of care, so similar behavior could be expected^22^. No comparative studies were found using the Prescrire criteria. It may be due to the specialized nature of the drugs comprised by this criterion, and the fact that it is not designed for a specific age group.

Of the three international criteria used, the STOPP criterion identified significantly higher number of PPI than the Beers, similarly to those identified in other studies^9,23^. However, identification is enhanced by combining criteria, as proposed in this study (BSP). This suggests that combined criteria may be a potentially better option for the identification of PPI.

A steady increase in prevalence was observed as the number of medications increased, with a maximum at 7 medications, similar to other studies in which an increased risk of PPI is related to each additional medication prescribed, with possibilities ranging from 2 to 6 times more with the intake of 6 or more medications^24,25^.

This study found differences in the prevalence of PPI by region, higher in the north of the country, and we could not attribute it to any study variable. To explain this, a comparison was made by proportion of AM in each state, as well as by the total number of chronic patients (diabetic and hypertensive), and no difference was found to explain this behavior. Another possible cause could be access to medications (availability or supply in the basic pharmacopoeia). This situation is not documented in this or other studies.

The NSAIDs were the most prescribed group of drugs among AM, as in other studies^21,26^. Despite the consensus that they should not be used in AM for the management of chronic pain, as they are associated with an increased risk of gastric ulcer and bleeding, worsening of cardiovascular pathologies and drug-drug interactions. On the other hand, not using these drugs represents a challenge for health personnel, since chronic pain in AM affects ambulation, with an increased risk of falls, complications associated with depression, cognitive deterioration and increased drug consumption^8,25^.

According to studies carried out among AM on chronic pain management, paracetamol continues to be the first choice for the management of mild to moderate pain, especially musculoskeletal pain, followed by meloxicam. Both medications should be used respecting the maximum recommended dose and taking into account its contraindication in users with hepatic dysfunction and severe renal insufficiency^27^. However, in Mexico, meloxicam is not included in the basic drug list^28^. So, the use of adjuvants is important when availability of drugs is limited. For example, it has been shown that physical therapy can be beneficial in some AM^27^.

Finding five groups of drugs for chronic pathologies among the ten most frequent PPIs is a consequence of their high prevalence in Mexico, where cardiovascular disease is the main cause of death in the population of AM, followed by diabetes mellitus^6^. Among other factors, the decrease in hepatic parenchyma, as well as of blood flow in AM, decreases the bioavailability of certain drugs, including some antihypertensive medications, which could lead the physician to prescribe higher doses, increasing the risk of adverse reactions^29^. On the other hand, the decrease in the glomerular filtration rate, and consequently the decrease in drug excretion, may cause frequent adverse events if the necessary dosage adjustments are not made.

Special care should be taken with the use of sulfonylureas for the treatment of diabetes mellitus. The risk of severe hypoglycemia is high, which can lead to more serious events such as myocardial infarction and cerebral vascular accidents. All oral hypoglycemic agents in AM should be indicated at the lowest effective dose to minimize these adverse events^19^, in addition to ensuring adequate follow-up of non-pharmacological treatment^30^.

As in other studies, antihistamines are an important group of PPIs, despite their association with increased morbidity, hospitalizations, cognitive impairment and mortality^25,29^. As these are medications that are not regularly chronically used, the search for alternatives is a feasible possibility for the service provider.

## CONCLUSIONS

This population-based study showed that the use of PPIs in AM, in users of public health services, is high in Mexico, implying that the quality of prescriptions is low. Although the prevalence of PPI is not attributable exclusively to health personnel, they are the ones who have the necessary knowledge to seek the best therapeutic alternative to limit the possibility of AE.

The AM presents comorbidities that often require management by different levels of care, as well as a greater number of medications, thus increasing the risk of PPI and, therefore, the risk of AE. This makes the management of these patients complex, increases costs for the health system and further fragments the possibility of comprehensive care due to the lack of coordination between physicians, and between levels of care.

Despite the growing number of AM worldwide and the correlation with multimorbidity, the multiple medical care guidelines and protocols are usually directed at a single condition, and frequently conflict with treatments for other diseases^31^. This increases the risk of PPI derived from the sum of drugs for a particular condition, which may be ideal if considered individually, but dangerous if patient is not assessed integrally, exposing them to pharmacological interactions and side effects that reduce their quality of life.

In the light of quality, it is necessary to develop studies with better and deeper analysis of the PPI tools, combining and adapting them to the country’s context, to be used as a guide. In addition, there is a need to improve regulation on the provision and prescription of medicines in synergy with the PPI tools, and favorable to the proper prescription of medicines.

### Limitations

The source of information used derives from a national survey, whose main objective was not aimed at screening for PPI. Therefore, there were limitations regarding the clinical information (morbidity and comorbidities) required to apply all the criteria of the PPI screening tools.

Dosage, frequency, and timing of prescribed medications were not considered as part of compliance with criteria.

Comparison of prevalence levels with other studies is difficult because each study has considered different populations and made adjustments based on the information available.
