# Supplementary material for: Potentially inappropriate prescribing in older adults in Mexico
Source: Rev Saude Publica. 2021 Nov 12;55:80. doi: 10.11606/s1518-8787.20210550033110 (PMC8601402; doi:10.11606/s1518-8787.20210550033110)
Supplement: Translation

# Translation: Prescripción potencialmente inapropiada en adultos mayores en México

**Authors:** Pedro Jesús Saturno-Hernández, Ofelia Poblano-Verástegui, Omar Acosta-Ruiz, Arturo Cuauhtémoc Bautista-Morales, Patricia María Gómez-Cortez, José Luis Alcántara-Zamora, Luis Miguel Gutiérrez-Robledo

**Affiliations:** I México Centro de Investigación en Evaluación y Encuestas. Instituto Nacional de Salud Pública. Morelos, México; II México Consorcio Mexicano de Hospitales Privados. Ciudad de México, México; III México Investigador independiente. Ciudad de México, México; IV México Instituto Nacional de Geriatría. Ciudad de México, México

**Keywords:** Anciano, Prescripción Inadecuada, Comorbilidad, Mal Uso de Medicamentos de Venta con Receta, Efectos Colaterales y Reacciones Adversas Relacionados con Medicamentos

## Abstract

**OBJETIVO:**

Identificar y cuantificar la prescripción potencialmente inapropiada (PPI) y otros problemas en la prescripción de medicamentos en los servicios públicos de atención médica en un estudio poblacional en los tres niveles de complejidad existentes en México.

**MÉTODOS:**

Análisis descriptivo del Estudio de Satisfacción de Usuarios del Sistema de Protección Social en Salud 2014–2016, sección de prescripción y surtimiento de medicamentos, para obtener la prevalencia de PPI en adultos mayores (≥ 65 años) con base en listados Beers, STOPP, Prescrire y BSP mediante indicadores de prescripción en AM, uno por cada listado.

**RESULTADOS:**

Al 67% de los AM se les prescribió al menos un medicamento, con una media de 2.7 medicamentos por receta. La prevalencia de PPI fue del 74% según los criterios BSP, del 67% según el listado STOPP, del 59% con los criterios Beer y del 20% con Prescrire. Las prescripciones PPI más frecuentes fueron los AINES, vasodilatadores y las sulfonilureas.

**CONCLUSIONES:**

El uso de PPI en AM es alto en México. La mayor prevalencia encontrada en este estudio puede ser reflejo de la utilización de una fuente con representatividad poblacional. La utilización parcial y adaptaciones de los criterios dificultan la comparabilidad entre estudios, sin embargo, los criterios STOPP son los que más altas prevalencias han presentado debido a que abarca un mayor número de medicamentos y su uso más habitual en el primer nivel de atención.

## INTRODUCCIÓN

La medicación en adultos mayores (AM) se reconoce como un problema de alta complejidad en la práctica clínica. Algunos de los factores que contribuyen a esta situación son los cambios fisiológicos asociados a la edad, dado que afectan la farmacodinamia y farmacocinética de algunos fármacos, así como la morbilidad múltiple que conlleva a variados regímenes farmacológicos. Además, influyen el envejecimiento poblacional y factores del sistema de salud como la limitada accesibilidad y actualización de lineamientos de consulta sobre farmacología en AM; atención con múltiples especialistas y frecuentemente en diferentes niveles de complejidad. Este grupo poblacional es muy vulnerable a la prescripción de alto riesgo, dentro de la que se encuentra la prescripción potencialmente inapropiada (PPI)^1,2^.

En los AM, la prescripción de alto riesgo se define como aquella que puede conducir a resultados clínicos adversos o que no se ajusta al uso correcto de medicamentos. Se han propuesto una variedad de indicadores para cuantificar la prescripción de alto riesgo en AM, entre otros: polifarmacia, prescripción potencialmente inapropiada y exposición a medicamentos de alto riesgo^3^.

La PPI en el contexto de las personas mayores se puede definir como la prescripción de “medicamentos o clases de medicamentos que generalmente deben evitarse en personas de ≥ 65 años porque son ineficaces o representan un riesgo innecesariamente alto para las personas mayores y una alternativa más segura está disponible”^4,5^.

La prescripción en AM es un componente esencial en la atención médica y su correcta aplicación es un problema de salud pública en todo el mundo debido a que es el grupo poblacional que presenta mayor consumo, con el consecuente incremento de eventos adversos (EA) por interacciones medicamentosas, que conlleva a impactos clínico y económico^6,7^.

Los métodos de detección de PPI iniciaron en los 90´s, el primero en diseñar y publicar una herramienta de detección fue Beers, esta consistía en una lista explícita de medicamentos identificados por un grupo de expertos cuya prescripción sería inapropiada. Se diseñó para AM residentes de hogares especializados para su cuidado en los Estados Unidos de América (EUA); hay una actualización de 2012 para uso en la atención ambulatoria de la población ≥ 65 años. La actualización cuenta con un listado de 53 grupos de medicamentos potencialmente inapropiados divididos en tres categorías para AM: los que se deben evitar; a evitar con ciertas patologías y los que deben ser usados con precaución^8^.

Algunos países fueron creando o adaptando la lista Beers. Un caso concreto son los criterios STOPP, desarrollados en Europa por expertos en farmacoterapia geriátrica para AM que viven en la comunidad, que incluyen un mayor número de grupos de medicamentos potencialmente inapropiados (65), divididos por aparatos y sistemas. Además, consideran otros criterios como comorbilidad, severidad de la patología por el que se indica el medicamento y duración de tratamiento^9^.

Francia desarrolló sus propios criterios, llamados “Prescrire”. Es un listado de 74 grupos de medicamentos que son más dañinos que beneficiosos en todas las indicaciones, es decir, para todos los grupos de edad y población general; estos criterios fueron creados mediante la búsqueda bibliográfica sistemática en lugar de usar un panel de expertos^10^.

En los países desarrollados, un 20% de la población tiene ≥ 60 años y se estima que será un 32% para 2050. En los países en desarrollo, se espera que la proporción de la población de 60 años o más aumente del 8% en 2005 al 20% para 2050^6^.

En un país de ingresos medios como México, se estimó en 2019 que la población de ≥ 65 años era de 9.5 millones, lo que representa 7.5% de la población total. En 2030, los AM serán 14 millones (10.2% de la población total)^11^.

Las principales causas de muerte en AM en México durante el 2017 fueron enfermedades cardiovasculares (27.1%); diabetes mellitus (16.6%); enfermedades crónicas degenerativas, como los tumores malignos (11.6%), y enfermedades cerebrovasculares (6.7%)^12^.

En EUA, utilizando los criterios de Beers, se encontró una prevalencia de PPI en AM atendidos ambulatoriamente del 25 al 30% y del 40% en estancias de atención al AM (2009 y 2012)^2,8^. Cifras similares se han presentado en Irlanda en 2008, el 25% usando los criterios Beers y el 35% con los criterios STOPP^9^. En México se han realizado pocos estudios, en 2014 en un hospital público se realizó un estudio de prevalencia de PPI en AM de ≥ 70 utilizando los criterios Beers encontrando prevalencias del 49%. Sin embargo, cada prevalencia reportada se debe tomar con reserva, por la alta variabilidad de las muestras seleccionadas, así como del uso parcial de los componentes de cada criterio para la medición^13^.

El uso inapropiado de medicamentos es una preocupación importante en la seguridad del paciente, especialmente para la población de AM, por sus características, como ya se mencionó previamente^6^. La PPI tiene importantes impactos en varias áreas de la atención a la salud. Se ha estudiado como la identificación de PPI en AM puede reducir la carga de EA, hospitalización y mortalidad por medicamentos^1,2,5^. Además se estimó un gasto anual en salud relacionado con PPI de 7.2 mil millones de dólares en EUA^8^.

México cuenta con una Lista de Medicamentos Esenciales (EDL)^14^ que sirve de guía para todas las instituciones públicas de salud, se basa en el concepto de uso racional de medicamentos propuesto por la Organización Mundial de la Salud (OMS): eficacia, seguridad y costo. Sin embargo, existen pocos estudios sobre patrones y errores de prescripción de medicamentos en AM en México y aún menos en instituciones públicas de atención primaria a la salud o atención ambulatoria^6^. En los últimos cinco años, se cuenta con dos estudios realizados en unidades de primer nivel de atención del Instituto Mexicano del Seguro Social, que es una de las principales instituciones de salud de México que atiende a la población con seguridad social, en ambos estudios, las poblaciones reportadas fueron pequeñas^15,16^. Dentro de los hallazgos en estos estudios está: el uso de antiinflamatorios no esteroideos en pacientes hipertensos, con enfermedad ácido péptica o por un periodo mayor a tres meses; uso de glibenclamida que aumenta el riesgo de hipoglucemias; uso de benzodiacepinas con riesgo de trastornos del equilibrio; uso de calcio-antagonistas en pacientes con estreñimiento; uso de diuréticos en pacientes sin insuficiencia cardiaca congestiva; β-bloqueadores combinado con verapamilo o en presencia de enfermedad obstructiva crónica a los cuales puede alterar la dinámica ventilatoria.

Es necesario dimensionar el problema de prescripción inapropiada de medicamentos a nivel poblacional, analizar su prevalencia y los medicamentos que con mayor frecuencia se prescriben de forma inapropiada. Así, podría plantearse la disminución del riesgo de eventos asociados a una mala prescripción, reducir costos y brindar una atención más segura y efectiva. El objetivo de este estudio es identificar y cuantificar la PPI y otros problemas en la prescripción de medicamentos en los servicios públicos de atención médica en un estudio poblacional en los tres niveles de complejidad existentes en México.

## MÉTODOS

Se realizó un estudio descriptivo de corte transversal del Estudio de Satisfacción de Usuarios del Sistema de Protección Social en Salud (ESASPSS) aplicado en 2014, 2015 y 2016. El objetivo general del ESASPSS fue documentar la percepción de satisfacción de las(los) usuarias(os) del SPSS respecto de los servicios de salud que recibían y, en su momento, realizar recomendaciones para incrementar la satisfacción de las(los) usuarias(os) e incidir en el acceso efectivo a los servicios de salud.

La encuesta fue probabilística, estratificada y bietápica con representatividad nacional, estatal y regional. Las unidades médicas a las que acudieron los usuarios fueron definidas como unidades primarias de muestra, el número aproximado de unidades por estado fue de 26, un total de 832 unidades aproximadamente en el país, distribuidas en los tres niveles de atención: el 80% primer nivel, el 16% segundo nivel y el 4% tercer nivel. Por cada unidad seleccionada se aplicaron 31 cuestionarios, estimando una muestra efectiva de alrededor de 26,000 entrevistas en todo el país por año. La descripción detallada del procedimiento de muestreo y metodología de la encuesta se encuentra descrita en una publicación previa^17^.

Para este estudio se utilizó la sección de prescripción y surtimiento de medicamentos de la ESASPSS de los tres años, que contiene información de los medicamentos registrados en las recetas de los usuarios. Se incluyó en el análisis a AM de ≥ 65 años de edad, que acudieron a los establecimientos de salud y se les prescribió al menos un medicamento el día de la encuesta. Se excluyeron los AM que acudieron por otros motivos que no fuera consulta médica, se obtuvo una submuestra final de 6,071 AM acumulada en los tres años.

Para la identificación de la PPI se usaron los listados Beers^8^, STOPP^9^ y Prescrire^10^. Para los criterios Beers se tomó la actualización de 2012, no se excluyó ninguno de los 53 grupos de medicamentos que integran este criterio. En los criterios STOPP se usó el listado de 2008. Para Prescrire, se tomó la versión 2016 de 74 medicamentos. A partir de los tres criterios, se creó un criterio llamado “BSP”, en el cual se considera PPI en los casos en que se encontraba al menos un medicamento contraindicado en cualquiera de las listas. Algunos medicamentos contraindicados aparecen en dos o más listados de los criterios anteriores.

Para los cuatro criterios, se consideraron la totalidad de los medicamentos enlistados con independencia del diagnóstico o condición de cada individuo en la base de datos.

### Construcción y Análisis de Indicadores

Para el análisis se construyeron cuatro indicadores, dos indicadores generales de prescripción en AM, uno sobre el porcentaje de PPI de acuerdo a cada criterio utilizado y, por último, uno que valora los grupos de medicamentos contraindicados más frecuentes (Cuadro).

**Cuadro:** Descripción de los indicadores de medicamentos contraindicados en adultos mayores (AM).

Indicador	Fórmula	Descripción
**I. Indicadores generales de prescripciones en AM**^a^		
Porcentaje de usuarios AM con receta	$\frac{\Sigma \;\mathrm{AM}\;\mathrm{con}\;\mathrm{receta}}{\Sigma \;\mathrm{Total}\;\mathrm{de}\;\mathrm{AM}}$	Proporción de AM a quienes se les prescribió un tratamiento farmacológico.
Promedio de medicamentos en la receta del AM	$\frac{\Sigma \;\mathrm{Medicamentos}\;\mathrm{prescritos}\;a\;\mathrm{AM}}{\Sigma \;\mathrm{AM}\;\mathrm{con}\;\mathrm{receta}}$	El número de medicamentos que en promedio tienen las recetas. Este indicador permite conocer un grado de polifarmacia que hay en la prescripción en unidades del Seguro Popular.
**II. Porcentaje de medicamentos contraindicados según criterios**^b^		
Porcentaje de medicamentos contraindicados prescritos en AM	$\frac{\Sigma \;\mathrm{Medicamentos}\;\mathrm{contraindicados}\;\mathrm{en}\;\mathrm{AM}}{\Sigma \;\mathrm{Medicamentos}\;\mathrm{en}\;\mathrm{la}\;\mathrm{receta}\;\mathrm{del}\;\mathrm{AM}}$	Según la clasificación, son los medicamentos contraindicados prescritos divididos por el número de medicamentos en la receta, si el valor es cercano a 1 es que la mayoría de los medicamentos en la receta están contraindicados, si tiende a 0 es que es la menor proporción de medicamentos contraindicados.
**III. Grupo de medicamentos contraindicados más frecuentes prescritos a AM**		
Porcentaje del grupo de medicamentos contraindicados más recetados	$\frac{\Sigma \;\mathrm{Grupo}\;\mathrm{contraindicado}\;\mathrm{en}\;\mathrm{AM}}{\Sigma \;\mathrm{Medicamentos}\;\mathrm{contraindicados}\;\mathrm{en}\;\mathrm{AM}}$	Según el tipo de medicamento se categorizan los medicamentos y se dividen por el total de contraindicados.

^a^ Se calcula el indicador estratificando por población de adultos mayores y población de no adulto mayor, para cada año que se realizó la encuesta.

^b^ Se calcula el estimador de la misma forma para cada uno de los criterios (Beers, STOPP, Prescrire y BSP), estratificando por el número de medicamentos que se prescriben al usuario en la receta.

Para la aplicación del indicador de PPI por criterio, se calificaron y codificaron los medicamentos prescritos de cada usuario, generando variables binomiales por criterio, se otorgó el valor de 1 = contraindicado y 0 = no contraindicado. El procedimiento y fórmulas utilizadas para cada indicador se detallan en el Cuadro.

Para comparar porcentajes entre años de la encuesta se utilizó la prueba de ji2 de Pearson, tomando como significativo un valor de p ≤ 0.05. Adicionalmente, el indicador se analizó por área geográfica, creando cuatro mapas de la República Mexicana (uno por cada criterio), donde se clasificó la prevalencia de PPI. Para asignar la tonalidad en todos los mapas, se categorizaron en quintiles del 20% según el rango (el máximo menos el mínimo) de cada criterio.

Por último, para determinar los principales grupos de PPI, se agruparon de acuerdo a la sustancia activa señalada en la página de la Food and Drug Administration de Estados Unidos (Administración de Medicamentos y Alimentos)^18^. Para la distribución de los grupos de medicamentos, se contabilizaron según la sustancia activa que contienen y se dividieron entre el grupo de medicamentos prescritos en AM, con su respectivo intervalo de confianza al 95%.

Todos los cálculos estadísticos se ajustaron por el diseño de la encuesta, considerando los ponderadores para obtener estimadores poblacionales.

## RESULTADOS

Del total de AM en los tres años de estudio (6,071), en promedio, el 67% de los AM recibieron prescripción de al menos un medicamento. En 2014 se presentó la prevalencia más alta de prescripción con el 71%, con un descenso de 13 puntos porcentuales en 2016. La media de medicamentos por receta fue de 2.7 (IC 95% 2.7, 2.8), la diferencia por año solo fue significativa en 2016 donde se presentó la media más baja con 2.4 medicamentos por receta (tabla 1).

**Tabla 1:** Resultados de indicadores generales de prescripciones en adultos mayores (AM), según el año de la encuesta.

Año	2014	2015	2016	Total
**Total de AM en la encuesta**	**2.814**	**2.023**	**1.234**	**6.071**
**Total de medicamentos prescritos en AM**	**1.996**	**1.352**	**710**	**4.058**
Porcentaje de AM con receta (95%IC)	70,8 (69,1–72,5)	66,8 (64,8–68,9)	57,4 (54,6–60,1)	66,8 (65,6–67,9)
Promedio de medicamentos por receta en AM (95%IC)	2,7 (2,6–2,8)	2,9 (2,8–2,9)	2,4 (2,3–2,5)	2,7 (2,7–2,8)
Porcentaje de medicamentos contraindicados en AM según criterio Beers^a^ (95%IC)	60,6 (58,5–62,8)	60,2 (57,6–62,8)	53,8 (50,1–57,5)	59,3 (57,8–60,8)
Porcentaje de medicamentos contraindicados en AM según criterio STOPP ^a^ (95%IC)	69,6 (67,6–71,6)	68,3 (65,9–70,8)	57,0 (53,4–60,7)	67,0 (65,5–68,4)
Porcentaje de medicamentos contraindicados en AM según criterio Prescrire^a^ (95%IC)	19,8 (18,1–21,6)	22,7 (20,5–24,9)	15,2 (12,6–17,9)	20,0 (18,8–21,2)
Porcentaje de medicamentos contraindicados en AM según criterio BSP* (95%IC)	**76,3 (74,4–78,2)**	**76,4 (74,1–78,7)**	**64,6 (61,1–68,2)**	**74,3 (73,0–75,6)**

^a^ Porcentaje calculado sobre el total de medicamentos prescritos en AM.

En la tabla 1 se observa la prevalencia de PPI en AM por cada criterio. La prevalencia más alta se presentó con el uso del criterio creado para el estudio “BSP” con 74% (IC 95% 73.0, 75.6), este comportamiento ocurrió en los tres años del estudio. De los criterios usados internacionalmente, STOPP fue el de mayor prevalencia, promedio del 67%, y la menor prevalencia fue Prescrire, promedio del 20%. En 2016 se presentaron las cifras más bajas para los cuatro criterios utilizados, con respecto a los años anteriores.

La prevalencia de PPI no presenta diferencias significativas entre años de medición y criterio utilizado, pero se observa un incremento de la prevalencia conforme aumenta el número de medicamentos prescritos (figura 1). En los criterios Beers, STOPP y BSP la mayor prevalencia se presentó cuando se recetaron 7 medicamentos en la misma receta (29.6%, 32.6% y 44.3% en promedio respectivamente).

**Figura 1:** Medicamentos contraindicados según número de medicamentos en la receta, por criterio y año.

En la figura 2 se observan las regiones del país con mayor prevalencia de PPI. En el norte prevalecen los porcentajes más altos usando los criterios Beers, STOPP y BSP; Chihuahua, Durango y Zacatecas fueron los mayores. En el resto de la república hay variabilidad entre criterios. En la región sur, Veracruz y Yucatán alcanzan prevalencias como las observadas en los estados del norte.

**Figura 2:** Porcentaje de medicamentos contraindicados prescritos en adultos mayores por entidad federativa en los años 2014–2016.

Los AINES son el grupo de PPI que más se prescribieron en la muestra de los tres años (25%, 36% y 32% respectivamente), en segundo lugar, se encuentra un grupo de hipoglucemiantes orales (sulfonilureas), seguido de medicamentos para patologías cardiovasculares como son: vasodilatadores, beta-bloqueadores, diuréticos y bloqueadores de calcio (tabla 2).

**Tabla 2:** Grupos de medicamentos contraindicados prescritos con mayor frecuenciaa en adultos mayores (AM).

2014		2015		2016		2014–2016	
**Tipo de medicamento**	**n = 2.539**	**Tipo de medicamento**	**n = 1.696**	**Tipo de medicamento**	**n = 673**	**Tipo de medicamento**	**n = 4.908**
AINES	25,0% (23,4–26,7)	AINES	26,4% (24,3–28,5)	AINES	31,8% (28,3–35,3)	AINES	26,4% (25,2–27,7)
Vasodilatadores	13,7% (12,3–15,0)	Vasodilatadores	11,1% (9,6–12,6)	Sulfonilureas	8,8% (6,6–10,9)	Vasodilatadores	12,0% (11,1–12,9)
Sulfonilureas	12,8% (11,5–14,1)	Sulfonilureas	10,8% (9,4–12,3)	Diuréticos	8,3% (6,2–10,4)	Sulfonilureas	11,6% (10,7–12,4)
Diuréticos	9,4% (8,3–10,5)	Diuréticos	9,1% (7,7–10,4)	Inhibidores de la bomba de protones	7,7% (5,7–9,7)	Diuréticos	9,1% (8,3–10,0)
Betabloqueadores	8,5% (7,4–9,6)	Betabloqueadores	9,0% (7,6–10,3)	Vasodilatadores	7,7% (5,7–9,7)	Betabloqueadores	8,5% (7,7–9,2)
Bloqueadores de canales de calcio	7,0% (6,0–8,0)	Bloqueadores de canales de calcio	7,1% (5,9–8,4)	Betabloqueadores	7,0% (5,1–8,9)	Bloqueadores de canales de calcio	7,0% (6,3–7,7)
Inhibidores de la bomba de protones	5,8% (4,9–6,7)	Inhibidores de la bomba de protones	5,4% (4,3–6,5)	Bloqueadores de canales de calcio	6,8% (4,9–8,7)	Inhibidores de la bomba de protones	5,9% (5,3–6,6)
Fibratos	3,3% (2,6–4,0)	Fibratos	4,4% (3,4–5,3)	Fibratos	4,6% (3,0–6,2)	Fibratos	3,8% (3,3–4,4)
Antagonista de receptor H2	2,8% (2,2–3,5)	Antagonista de receptor H2	3,1% (2,2–3,9)	Corticoesteroides	2,2% (1,1–3,3)	Antagonista de receptor H2	2,8% (2,3–3,3)
Antihistamínicos	2,4% (1,8–3,0)	Antihistamínicos	2,2% (1,5–2,9)	Antagonista de receptor H2	2,1% (1,0–3,2)	Antihistamínicos	2,3% (1,9–2,7)

^a^ Valores en porcentaje (IC95%).

## DISCUSIÓN

Los resultados de este estudio muestran aspectos relevantes en la calidad de la prestación de servicios de salud a la población de ≥ 65 años, en particular con relación a la prescripción de medicamentos. Por tipo de criterios utilizados existen diferencias por región, tipo de medicamento y número de medicamentos recetados. Debido a que la mayor proporción de la muestra se encuentra representada por unidades médicas de primer nivel de atención, los resultados muestran principalmente el comportamiento de estas unidades.

El número de medicamentos por receta está por debajo de lo encontrado por la OMS (3.5) en el mismo grupo de edad de la población^19^. A pesar de que el número de medicamentos es menor, la prevalencia de PPI encontrada en el estudio es similar a la reportada por estudios comunitarios bajo los criterios STOPP, realizados en Canadá, Europa, Australia y Asia (del 21% al 69%)^20^. El intervalo entre las prevalencias, muy amplio, puede derivar del uso indistinto de múltiples criterios, adaptaciones, y de la fuente de información utilizada para la aplicación de los mismos, en especial información sobre dosificación y el historial de patologías de la población en estudio. Lo anterior dificulta la comparabilidad entre estudios.

En 2014, estudios poblacionales realizados en Irlanda, en AM de ≥ 65 años, encontraron prevalencias del 15% tomando en cuenta la morbilidad y dosificación de los medicamentos y del 36% tomando solo la presencia del medicamento contraindicado. Para ambos estudios se utilizaron los criterios STOPP y la muestra fue similar al presente estudio^21^. Las prevalencias de los estudios anteriores son dos veces menores que lo encontrado en este estudio, esta diferencia puede ser resultado del uso de la comorbilidad para la identificación de los PPI; simplificar el criterio sin considerar esta variable permite que mayor número de medicamentos se consideren PPI, elevando las prevalencias. En un estudio realizado en una unidad de primer nivel de atención en México, en el cual se consideró la morbilidad, se encontró una prevalencia del 67%^15^, igual a la encontrada en este estudio, por lo que se podría inferir que, la prevalencia de PPI en México es alta.

En Latinoamérica, Colombia y Argentina específicamente, se han encontrado prevalencias más bajas (el 21% y el 20% respectivamente) usando los criterios Beers. En estos estudios solo consideraron la atención ambulatoria de primer nivel de atención en AM; nuestros resultados tienen una mayor representatividad del primer nivel de atención por lo que se podría esperar un comportamiento similar^22^. No se encontraron estudios comparativos en los que se usaran los criterios Prescrire, esto puede ser por lo especializado de los medicamentos que contiene este criterio y que no está diseñado para un grupo de edad específico.

De los tres criterios internacionales usados, el criterio STOPP identificó significativamente mayor número de PPI que Beers, similar a los identificados en otros estudios^9,23^. Sin embargo, la identificación se potencia al realizar una combinación de criterios como es el propuesto en este estudio (BSP), lo que indica el potencial para que los criterios combinados sean usados como una mejor opción para la identificación de PPI.

Se observó un incremento constante de la prevalencia conforme se incrementó el número de medicamentos, con un máximo a los 7 medicamentos, similar a otros estudios en los que se relaciona un mayor riesgo de PPI con cada medicamento adicional recetado, con posibilidades que van desde 2 a 6 veces más con la toma de 6 o más medicamentos^24,25^.

En este estudio se observaron diferencias en la prevalencia de PPI por región, más alta en el norte del país, sin poder atribuirlo a alguna variable del estudio. Para explicarlo se realizó la comparación por proporción de AM en cada estado, así como, por el total de pacientes crónicos (diabéticos e hipertensos), sin encontrar diferencias que pudieran explicar este comportamiento. Otra posible causa sería el acceso a los medicamentos (disponibilidad o abasto en el cuadro básico), situación que no está documentada en este ni en otros estudios.

Los AINES fueron el grupo de medicamentos más recetado en AM, igual que en otros estudios^21,26^. A pesar del consenso de no usarse en AM para manejo del dolor crónico, por estar asociado a incremento del riesgo de úlcera y hemorragia gástrica, empeoramiento de patologías cardiovasculares e interacciones medicamentosas. Por otro lado, no utilizar estos fármacos representa un reto para el personal de salud, ya que el dolor crónico en AM afecta la deambulación, con incremento en el riesgo de caídas, complicaciones asociadas con depresión, deterioro cognitivo y mayor consumo de medicamentos^8,25^.

De acuerdo a estudios realizados en AM sobre el manejo del dolor crónico, el paracetamol sigue siendo la primera elección para el manejo de dolor leve a moderado, en especial el osteomuscular; en segundo lugar, el meloxicam, ambos deben ser usados respetando la dosis máxima recomendada y tomando en cuenta su contraindicación en usuarios con disfunción hepática e insuficiencia renal grave^27^. Sin embargo, en México, el meloxicam no se encuentra en el cuadro básico de medicamentos^28^, por lo que el uso de adyuvantes es importante cuando se tienen limitaciones de disponibilidad de medicamentos, se ha demostrado que, por ejemplo, la terapia física puede ser beneficiosa en algunos AM^27^.

Encontrar cinco grupos de medicamentos para patologías crónicas entre los diez más frecuentes de PPI, es una consecuencia de su alta prevalencia en México, donde la enfermedad cardiovascular es la principal causa de muerte en la población de AM, seguida por la diabetes mellitus^6^. Entre otros factores, la disminución del parénquima hepático, así como del flujo sanguíneo en AM, disminuye la biodisponibilidad de ciertos fármacos, entre ellos algunos antihipertensivos, podría generar que el médico prescriba mayores dosis, lo que elevaría el riesgo de reacciones adversas^29^. Por otra parte, la disminución del filtrado glomerular, y por consecuencia la disminución de la excreción de fármacos, causa frecuentes eventos adversos si no se realizan los ajustes necesarios en la dosificación.

Se debe tener especial cuidado con el uso de sulfonilureas para el tratamiento de diabetes mellitus, el riesgo de hipoglucemia severa es alto, la cual puede conllevar a eventos de mayor gravedad como infartos al miocardio y accidentes vasculares cerebrales. Todos los hipoglucemiantes orales en AM deben ser indicados con la menor dosis efectiva para minimizar estos eventos adversos^19^, además de asegurar un adecuado seguimiento del tratamiento no farmacológico^30^.

Al igual que en otros estudios, los medicamentos antihistamínicos son un grupo importante de PPI, a pesar de su asociación con aumento de la morbilidad, hospitalizaciones, deterioro cognitivo y mortalidad^25,29^. Al ser medicamentos que regularmente no se usan de forma crónica, la búsqueda de alternativas es una posibilidad viable para el prestador de servicios.

## CONCLUSIONES

Este estudio realizado a nivel poblacional evidenció que el uso de PPI en AM, en usuarios de Servicios de Salud Públicos, es alto en México, esto implica que la calidad de las prescripciones que se realizan es baja. A pesar de que la prevalencia de PPI no es solo responsabilidad del personal de salud, son ellos los que tienen los conocimientos necesarios para buscar la mejor alternativa terapéutica que limite la posibilidad de EA.

El AM presenta comorbilidades que requieren en muchas ocasiones el manejo por distintos niveles de atención, así como un mayor número de medicamentos, lo cual incrementa el riesgo de PPI y esta a su vez de EA. Lo anterior vuelve complejo el manejo de estos pacientes, incrementa los costos para el sistema de salud y fragmenta aún más la posibilidad de su atención integral por la falta de coordinación entre médicos y entre niveles.

A pesar del creciente número de AM en todo el mundo y su correlación con multimorbilidad, las múltiples guías y protocolos de atención médica suelen estar dirigidas a una sola afección y frecuentemente contradicen los tratamientos para otras enfermedades^31^. Lo anterior incrementa el riesgo de PPI derivado de la suma de medicamentos para una afección en particular, que en lo individual puede resultar ideal, pero peligrosa si no se valora al paciente integralmente, exponiéndolo a interacciones farmacológicas y efectos secundarios que reducen su calidad de vida.

Desde la perspectiva de la calidad, es necesario el desarrollo de estudios con un mejor y profundo análisis de las herramientas de PPI, realizando combinaciones de ellas, y adecuándolas al contexto del país para ser utilizadas como guía. Además de la necesidad de mejorar la regulación de la disposición y prescripción de medicamentos que haga sinergia con las herramientas de PPI y favorezcan la apropiada prescripción de medicamentos.

### Limitaciones

La fuente de información utilizada deriva de una encuesta nacional, cuyo objetivo principal no estaba dirigido a la exploración de PPI, por lo que se tuvieron limitantes de información clínica (morbilidad y comorbilidades) necesarias para aplicar todos los criterios de las herramientas de detección de PPI.

No se consideró la dosificación, la frecuencia y el tiempo de los medicamentos prescritos como parte del cumplimiento de los criterios.

Es difícil la comparación de prevalencias con otros estudios, debido a que cada estudio ha considerado poblaciones distintas y ha realizado ajustes con base en la disponibilidad de la información.
